# Pleiotropic Role of Rainbow Trout CXCRs in Response to Disease and Environment: Insights from Transcriptional Signatures and Structure Analysis

**DOI:** 10.3390/biom14030337

**Published:** 2024-03-12

**Authors:** Zhi-Shuai Hou, Hong-Kui Zhao, Pedro Perdiguero, Meng-Qun Liu, Kai-Wen Xiang, Chu Zeng, Zhao Li, Xiao-Dong Yang, Qian Yang, Yuan-Ru Xin, Ji-Fang Li, Carolina Tafalla, Hai-Shen Wen

**Affiliations:** 1 Key Laboratory of Mariculture (Ocean University of China), Ministry of Education (KLMME), Ocean University of China, Qingdao 266003, China; 2 Animal Health Research Center (CISA-INIA-CSIC), 28130 Valdeolmos, Spain; 3 Department of Genetics, Physiology and Microbiology, Faculty of Biological Sciences, Complutense University of Madrid (UCM), 28040 Madrid, Spain

**Keywords:** rainbow trout, chemokine receptors, bacterial infection, environmental changes

## Abstract

Chemokines are cytokines with chemoattractant capacities that exert their physiological functions through the binding of chemokine receptors. Thus, chemokine and receptor complexes exert important roles in regulating development and homeostasis during routine immune surveillance and inflammation. Compared to mammals, the physiology and structure of chemokine receptors in fish have not been systematically studied. Furthermore, the salmonid-specific whole genome duplication has significantly increased the number of functional paralogs of chemokine receptors. In this context, in the current study, trout exhibited 17 *cxcr* genes, including 12 newly identified and 5 previously identified receptors. Interestingly, gene expression of brain *cxcr1* and *cxcr4*, kidney *cxcr3* and *cxcr4*, and spleen *cxcr3*, *cxcr4*, and *cxcr5* subtypes were altered by bacterial infection, whereas brain *cxcr1*, kidney *cxcr1* and *cxcr7*, and liver *cxcr2*, *cxcr3*, and *cxcr4* subtypes were changed in response to environmental changes. Based on protein structures predicted by ColabFold, the conserved amino acids in binding pockets between trout CXCR4.1 subtypes and human CXCR4 were also analyzed. Our study is valuable from a comparative point of view, providing new insights into the identification and physiology of salmonid chemokine receptors.

## 1. Introduction

Chemokines are small (8–15 kDa) proteins belonging to the cytokine family [1]. Chemokines bind to G protein-coupled receptors (GPCRs), and complexes of chemokine and receptor regulate cell movement and activation [2]. Based on the number and position of highly conserved N-terminal cysteines, chemokines are divided into four groups: CXC, CC, C, and CX3C (C indicates cysteine, and X/X3 indicates one or three non-cysteine amino acids) [1,2]. For example, CCL2 represents a chemokine ligand of the CC subfamily, number 2, and CCR2 represents the receptor of CCL2 [2,3]. It is well known that the physiological function of chemokines is to modulate cell migration, which gives them their name (from ‘chemotactic cytokines’) [4]. Chemokines play an important role in regulating cellular migration during routine immune surveillance, inflammation, and development [2]. Based on their physiological functions, chemokines can also be divided into two groups: inflammatory and homeostatic chemokines [5]. Inflammatory chemokines are induced directly by inflammatory stimuli or related cells [4,5]. Homeostatic chemokines, on the other hand, are involved in cell migration, organogenesis, and development, and they are constitutively expressed in discrete tissues or cells [4,5].

Chemokine receptors have also been divided into four groups: CXC, CC, C, and CX3C chemokine receptors, which are consistent with the four groups of chemokines [4]. Although a large number of chemokines have been identified, the number of chemokine receptors is lower [2,6,7]. For example, the human chemokine superfamily currently contains ~46 chemokines, and these chemokines bind to 18 chemokine receptors (six CXCRs, ten CCRs, one XCR, one CX3CR) [4]. Members of the chemokine superfamily (including ligands and receptors) have been identified in chicken, zebrafish, shark, and jawless fish [4].

Compared to mammals, teleosts exhibit increased gene copies of many immune genes, including chemokines, as a result of the teleost-specific whole genome duplication (which is also referred to as the third round of genome duplication (3R)) [8,9]. Hence, in 1998, the first teleost chemokine gene was identified in salmonids, and since then, a great number of chemokine orthologues, with a great complexity in physiology, have been identified in teleost, possibly also because chemokines are thought to evolve faster than other genes associated with immunomodulation [10,11,12]. On the other hand, previous studies in model animals showed that the CC chemokine receptor family contains at least 17 members in zebrafish (*Danio rerio*) and 10 members in medaka (*Oryzias latipes*) [13,14]. In aquacultured fish, 23 CC and 8 CXC chemokine receptors have been identified in channel catfish (*Ictalurus punctatus*) and 19 CC and 8 CXC chemokine receptors in orange-spotted grouper (*Epinephelus coioides*) [15,16]. Considering that chemokine receptors also influence how chemokines regulate immune development, homeostasis, and competence, the identification of the complete repertoire of chemokine receptors in teleost and the assessment of their functions will provide insights into the functionality of chemokines from a comparative point of view, thus contributing to boosting the immune response of fish for a sustainable development of aquaculture.

Rainbow trout (*Oncorhynchus mykiss*) belongs to the salmonid family and is one of the most studied teleost species, having been extensively used as a model in diverse research fields, including ecology, physiology, toxicology, immunology, and microbiology [17,18,19]. Rainbow trout is also an economically important aquacultured species with a global production of ~1,000,000 tons (FAO, 2022). In the trout industry, diseases caused by pathogen infection are of major ecological and commercial relevance to aquaculture. On the other hand, aquaculture and other anthropogenic activities might result in short-term and long-term changes in the natural aquatic environment. These environmental changes could provoke an important impact on fish physiology, including the immune system, thus having consequences on their well-being and disease resistance [20,21]. Interestingly, an additional round of whole genome duplication occurred in salmonid ancestors (which is referred to as the fourth round of genome duplication (4R) or salmonid-specific whole genome duplication) [18,22,23,24,25]. Duplicated copies of functional genes have been retained after this additional salmonid-specific whole genome duplication when compared to species that have only experienced the teleost-specific whole genome duplication [26,27,28]. These paralogs exhibit differences in sequence, transcription, and function [22,28,29,30].

In this context, the first aim of this study was to identify the complete repertoire of full-length CXC chemokine receptor genes in rainbow trout by exploiting the whole genomic data. We investigated the basal expressions of CXC chemokine receptors, as well as how they were transcriptionally regulated in response to *Vibrio anguillarum* and *Aeromonas salmonicida* infection. *Vibrio anguillarum* and *Aeromonas salmonicida* are two major pathogens that cause severe fatal diseases and considerable economic losses in cultured rainbow trout [31,32,33]. Environmental changes also impact fish immune systems by deregulating chemokine signaling in teleost [34,35], with triploid trout exerting different biochemistry and physiology when compared to diploid trout [36,37]. Therefore, we also investigated the transcriptional profiles of these CXC chemokine receptors in responses to environment changes in rainbow trout.

## 2. Materials and Methods

### 2.1. Ethics Statement

Our experiments were approved by the Institutional Review Board at Ocean University of China (permit number: 20141201) and performed in accordance with the U.K. Animal Scientific Procedures (Act, 1986) and associated guidelines, the EU Directive 2010/63/EU for animal experiments and the National Institutes of Health Guide for the Care and Use of Laboratory Animals use of laboratory animals (NIH Publications No. 8023, revised 1978). This study did not involve endangered or protected animals.

### 2.2. Genome-Wide Identification and Sequence Analyses

To identify the CXCR genes of rainbow trout, we searched the whole genome of rainbow trout obtained from NCBI (http://www.ncbi.nlm.nih.gov/, accessed on 24 January 2024) and performed tblastn analysis using all available CXCR sequences in the genome databases of human (Homo sapiens), mouse (*Mus musculus*), zebrafish (*Danio rerio*), Atlantic salmon (*Salmo salar*), fugu (*Takifugu rubripes*), Northern pike (*Esox lucius*), and channel catfish (*Ictalurus punctatus*) available in the NCBI (http://www.ncbi.nlm.nih.gov/), Ensembl (http://www.ensembl.org, accessed on 24 January 2024), and Uniport (http://www.uniprot.org/, accessed on 24 January 2024) as queries with e-values of 1 × 10^−5^. To remove redundant sequences, we used ClustalW for multiple alignments. Tandem arrangement genes were identified by their locations in the reference genome. The coding sequences were predicted using ORF (opening reading frames) finder (http://www.ncbi.nlm.nih.gov/gorf/gorf.html, accessed on 24 January 2024), which were further validated by BLASTP against NCBI nonredundant (nr) protein database. In addition, we used the online ProtParam tool to characterize the molecular weight (MW) and theoretical isoelectric point (pI).

Based on the amino acid sequences of CXC chemokine receptors of humans, mice, zebrafish, Atlantic salmon, medaka, fugu, Northern pike, and channel catfish, a phylogenetic analysis was conducted with MEGA 7, using the neighbor-joining method, with a set of 1000 bootstrap replicates [38].

### 2.3. Gene Structure, Conserved Domains, and Motif Analysis of the CXCR

Gene exon–intron structures were analyzed using the Gene Structure Display Server (GSDS2.0) by comparing the codon sequences and genomic sequences of the 17 CXCR members. The transmembrane (TM) domains were predicted by the TMHMM Server v. 2.0 (http://services.healthtech.dtu.dk/service.php?TMHMM-2.0, accessed on 24 January 2024), comparing the results of previous studies in human and zebrafish GPCRs. Motif analyses were performed with Multiple EM for Motif Elicitation (MEME, version 4.11.4), with the limitation of ten motifs and optimum widths of motifs of 6–50 amino acids [39].

### 2.4. Expression Analysis Using Available RNA-Seq Datasets

Using our available RNA-Seq datasets, we analyzed the cxcr expression levels of rainbow trout in response to bacterial infection (phenotype/timeline-specific expressions). The RNA-Seq datasets were retrieved from our previous studies described above:

1. Brain, kidney, and spleen samples from rainbow trout challenged with *Vibrio anguillarum* (SRA ID: PRJNA667799 [40,41,42]). Brain, kidney, and spleen samples were collected from control, asymptomatic, and symptomatic rainbow trout after *V. anguillarum* challenge, and 27 libraries of RNA-Seq samples were used (3 phenotypes × 3 tissues × 3 replicates [40,42]).
2. Brain and kidney samples from rainbow trout were challenged with *Aeromonas salmonicida* ([43]). Brain and kidney samples were collected from control and infected rainbow trout, and the RNA-Seq dataset included 12 libraries (2 (control vs. infection) × 2 tissues × 3 replicates [43]).
3. Brain, kidney, and liver samples from rainbow trout with environmental salinity changes ([44]). Diploid and triploid trout were classified into diploid trout in freshwater (DF), diploid trout in saltwater (DS, at salinity of 15 parts-per-thousand (ppt)), triploid trout in freshwater (TF), and triploid trout in saltwater (TS, at salinity of 15 ppt). Brain, liver, and kidney samples were collected from DS, TS, and TF. Twenty-seven libraries of RNA-Seq samples were used (3 groups (TF, DF, DS) × 3 tissues × 3 replicates [44]).
4. Liver samples from rainbow trout cultured in different stocking densities (unpublished data and count data are shown in Supplementary Materials). Rainbow trout were cultured in saltwater with initial densities at 9.15 kg/m^3^ (low density (LD)), 13.65 kg/m^3^ (moderate density (MD)), and 27.31 kg/m^3^ (high density (HD)) for 84 days. The final densities were 22.00 (LD), 32.05 (MD), and 52.24 (HD) kg/m^3^, respectively. Liver samples were collected from LD, MD, and HD on day 84.

### 2.5. Structural Analysis of Trout CXCR4.1 Subtypes

ColabFold (ColabFold v1.5.5) was used to predict the protein structures by combining MMseqs2 with AlphaFold2 or RoseTTAFold [45]. Compared to the ORF sequences, we showed amino acid sequences associated with TM, extracellular (ECL), and intracellular (ICL) loops with high confidence values. The amino acid sequences for structure prediction are shown in Supplementary Materials Text S1. The human CXCR4 (PDB ID: 4RWS) was used as a template. Comparison of the domains between trout and human CXCR4 and the cartoon, stick, and sphere structures of the proteins were generated by PyMOL software (PyMOL-2.5.4) [46,47].

### 2.6. Statistical Analysis

The RNA-Seq data (counts) were normalized with the Bioconductor DESeq2 Package [48,49]. In order to obtain the belt data (Poisson) distribution for further statistical analysis, data of RNA-Seq were normalized by log transformation [50]. After that, the normalized data were analyzed by an online R software Package (https://omicsforum.ca/, accessed on 24 January 2024) for multivariate analyses [51,52]. Based on previous studies in the fishery and biomedical studies [53,54], we evaluated the whole profile of the cxcr expressions by performing the heatmap, principal components analysis (PCA), correlation coefficients, and variable importance in projection (VIP). Gene expression analyses were performed with GraphPad Prism 8.0. The results were evaluated by one-way analysis of variance (ANOVA) followed by a Tukey multiple range test, with *p* < 0.05 set to assign significant differences. Student’s *t*-test was used for comparisons between two groups, with significance established when *p* < 0.05. Results were presented as mean ± standard error of the mean (SEM).

## 3. Results

### 3.1. Identification and Annotation of cxcr Genes in Rainbow Trout

In our study, a total of 17 *cxcr* genes (12 newly identified and 5 previously identified receptors) were identified in the rainbow trout, with the predicted protein sequences ranging from 309 to 461 amino acids, the molecular weights ranging from 33.92 to 50.85 kDa, and the pIs ranging between 5.88 and 9.24 (Table 1). Based on the sequence information of identified receptors in humans, mice, and zebrafish, on sequence similarities among the trout receptors, and on the conserved seven transmembrane domains and DRY motif, the 17 *cxcr* genes were divided into seven families. Chromosomal locations of *cxcr* genes were also studied. In brief, the trout *cxcr* genes were distributed in eight different chromosomes (Chr2, 3, 8, 16, 18, 22, 24, and 28), including three genes on Chr2, five genes on Chr3, and four genes on Chr22. Copy numbers of the *cxcr* genes in rainbow trout were compared with those of human, mouse, chicken, zebrafish, and several teleost species (Table 2). Expanded copies of *cxcr1*, *cxcr2*, *cxcr3*, *cxcr4*, and *cxcr7* genes were identified in rainbow trout (Table 2).

### 3.2. Phylogenetic Analysis and Gene Structure Analyses

**Figure 1:**
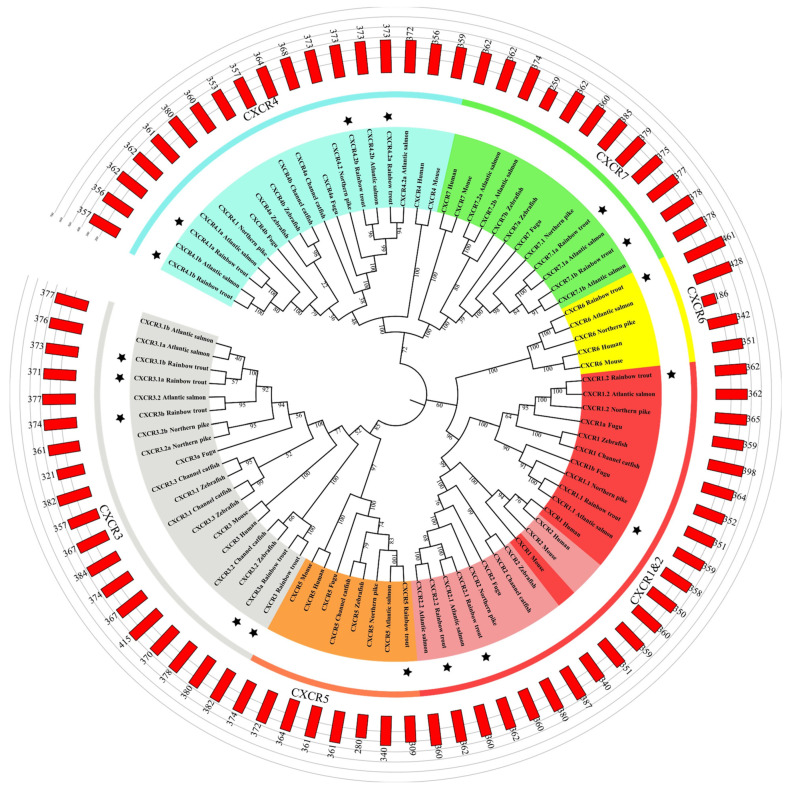
Phylogenetic tree of CXCRs. The CXCR sequences were obtained from rainbow trout, Atlantic salmon, zebrafish, human, mouse, Northern pike, Channel catfish, and fugu. The number of nodes shows the bootstrapping values, and the black stars indicate trout CXCRs.

**Figure 2:**
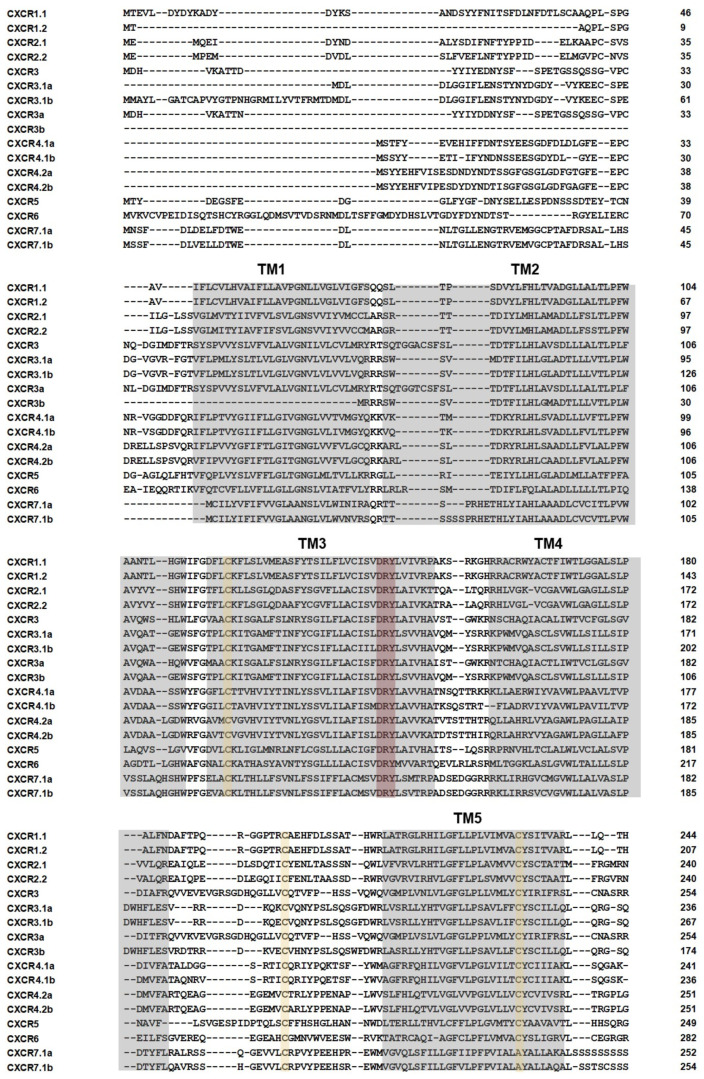

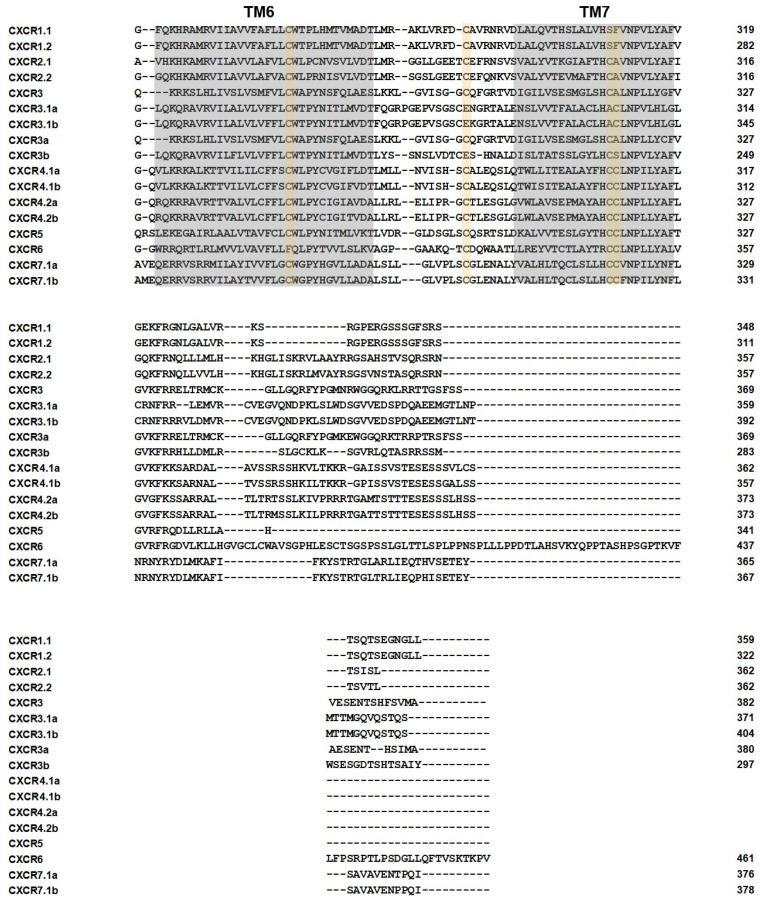
Alignment of CXCR proteins. Residues of transmembrane domains (TMs), DRY motif (a highly conserved motif in family A GPCRs), and (semi)conserved cysteines are shaded in grey, red, and orange. TMs and (semi)conserved cysteines are defined from previous studies of salmon and teleost CXCRs [61,62]. The highly conserved NPxxY motif in family A GPCRs is observed in TM 7.

Amino acid sequences of CXCR in rainbow trout and other species were used to construct a phylogenetic tree. The phylogenetic tree exerted a total of six subgroups, including the CXCR1, CXCR2, CXCR3, CXCR4, CXCR5, CXCR6, and CXCR7 subgroups (Figure 1). All these CXCR proteins contained seven transmembrane domains, which are typically observed in GPCRs (Figure 2).

### 3.3. Transcriptional Profiles of cxcr in Trout after Bacterial Infection

#### 3.3.1. *V. anguillarum*

**Figure 3:**
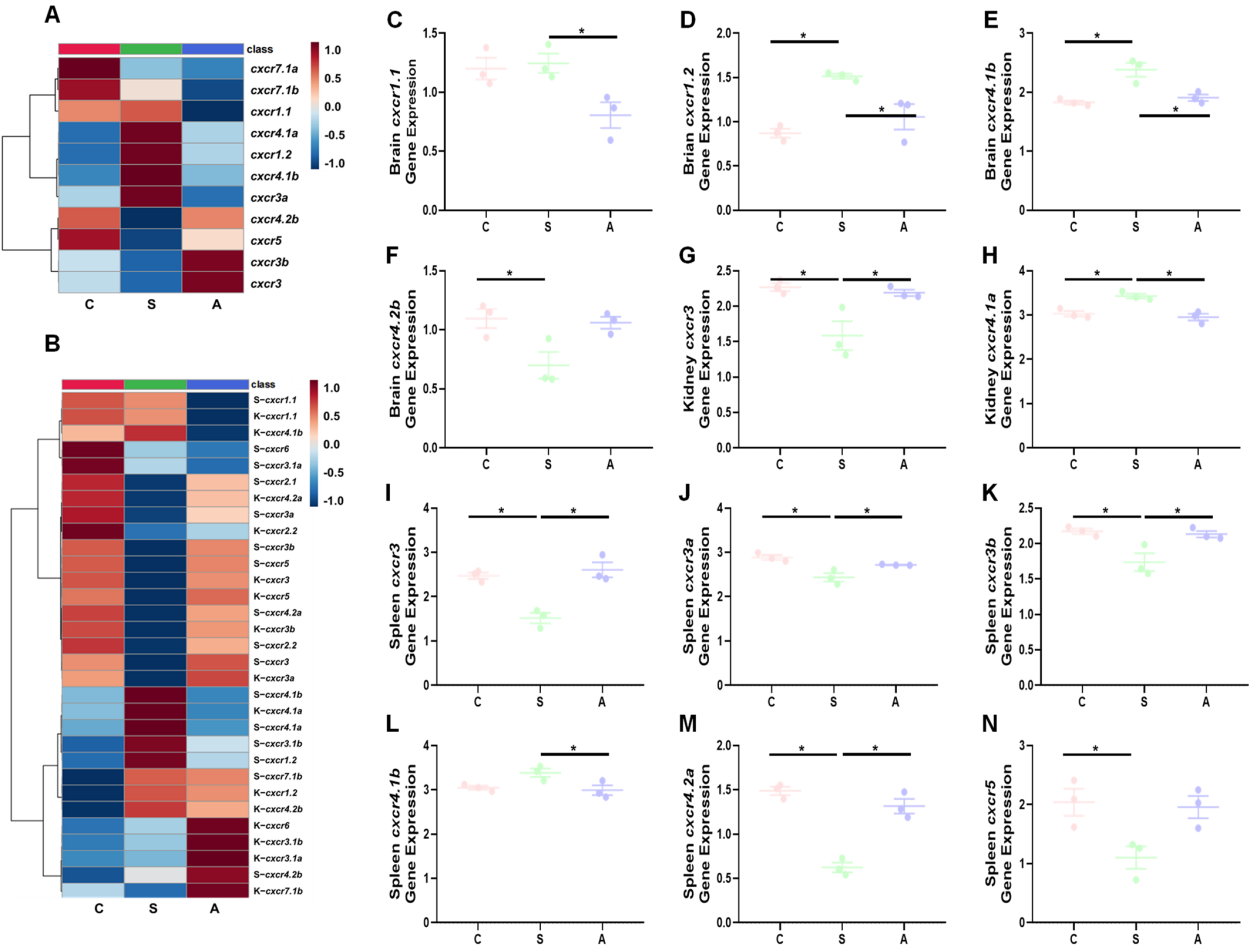
Transcriptional profiles of *cxcr* in trout after *V. anguillarum* infection. (**A**,**B**): the heatmap of *cxcr* transcriptional profiles ((**A**): brain; (**B**): kidney and spleen). (**C**–**N**): expression of the representative genes (with significant differences among groups). Asterisks indicate significant differences (one-way analysis ANOVA followed by Tukey’s multiple comparison test with *p* < 0.05). Abbreviations: K—kidney; S—spleen; C—control trout; A—asymptomatic trout; S—symptomatic trout.

The heatmap showed the overall expression profiles of *cxcr* genes in the brain (Figure 3A), spleen, and kidney (Figure 3B). Brain *cxcr1* and *cxcr4*, kidney *cxcr3* and *cxcr4*, and spleen *cxcr3*, *cxcr4*, and *cxcr5* subtypes were significantly altered by *V. anguillarum* infection (Figure 3C–N).

#### 3.3.2. *A. salmonicida*

**Figure 4:**
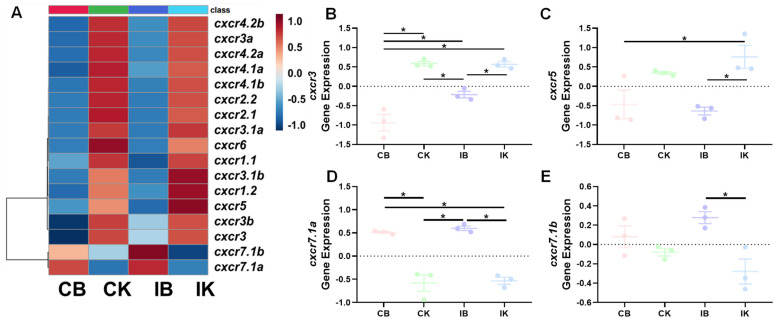
Transcriptional profiles of *cxcr* in trout after *A. salmonicida* infection. (**A**): the heatmap of *cxcr* transcriptional profiles. (**B**–**E**): expression of the representative genes (with significant differences among groups). Asterisks indicate significant differences (one-way analysis ANOVA followed by Tukey’s multiple comparison test with *p* < 0.05). Abbreviations: CB—control trout brain tissue; CK—control trout kidney tissue; IB—infected trout brain tissue; IK—infected trout kidney tissue.

The heatmap indicated the overall expression profiles of *cxcr* genes in the brain and kidney between control and infected trout (Figure 4A). Expressions of representative genes (*cxcr3*, *cxcr5*, *cxcr7.1a*, and *cxcr7.1b*) are also shown (Figure 4B–E).

### 3.4. Transcriptional Profiles of cxcr in Trout in Response to Salinity Change and High Stocking Density

**Figure 5:**
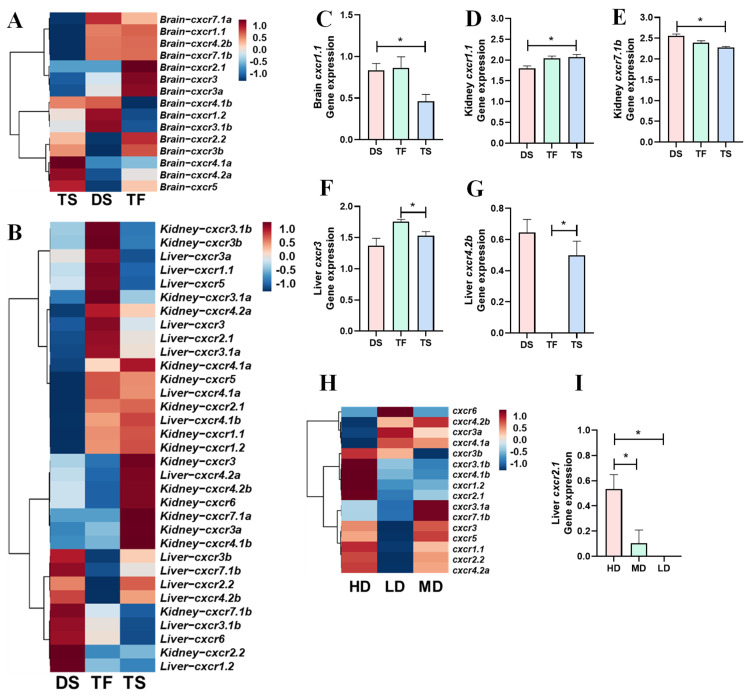
Transcriptional profiles of *cxcr* in trout after salinity and density changes. (**A**,**B**): principal component analysis (PCA) of *cxcr* transcriptional profiles in the brain (**A**) and liver and kidney (**B**). The separated PCA plots indicate specific *cxcr* transcriptions. (**A**,**B**): the heatmap of *cxcr* transcriptional profiles in the brain (**A**) and liver and kidney (**B**). (**C**–**G**): expression of the representative genes (with significant differences) between DS and TS or TF and TS (Student’s *t*-test was used for comparisons between two groups with *p* < 0.05). (**H**): the heatmap of *cxcr* transcriptional profiles in the liver. (**I**): expression of liver *cxcr1.2* among different stocking densities. Asterisks indicate significant differences (one-way analysis ANOVA followed by Tukey’s multiple comparison test with *p* < 0.05). Abbreviations: DS—diploid trout in saltwater; TF—triploid trout in freshwater; TS—triploid trout in saltwater.

The overall transcriptional profiles of trout *cxcr* in the brain, kidney, and liver are shown in heatmaps (Figure 5A,B). Compared to DS, TS showed a significantly down-regulated expression of brain *cxcr1.1*, up-regulated expression of kidney *cxcr1.1*, and down-regulated expression of kidney *cxcr7.1b* (Figure 5C–E). Compared to TF, TS showed a significantly down-regulated expression of liver *cxcr3* and up-regulated expression of liver *cxcr4.2b* (Figure 5F,G). The overall hepatic *cxcr* expressions were clustered in a heatmap (Figure 5H). High stocking density significantly increased *cxcr2.1* expressions when comparing HD to MD and LD (Figure 5I).

### 3.5. Structure Prediction of CXCR4.1a and CXCR4.1b

**Figure 6:**
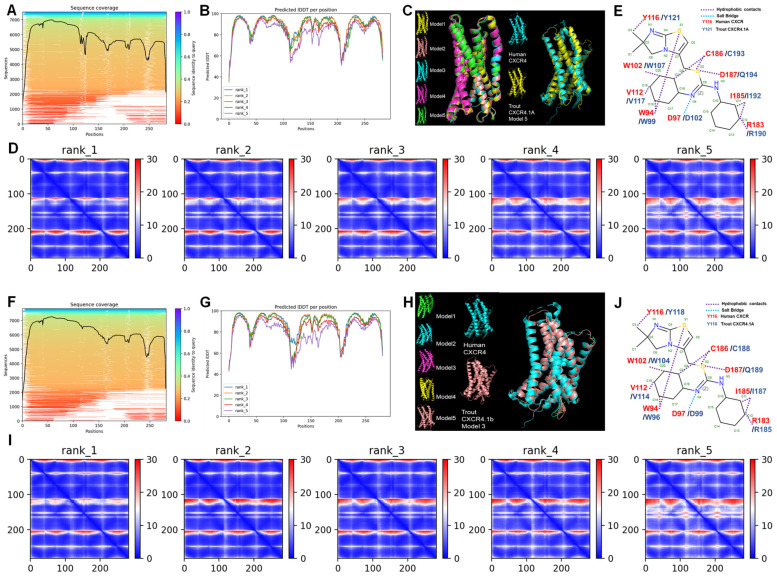
Comparison analyses of trout CXCR4.1a and CXCR4.1b based on human CXCR4 crystal structure. (**A**): sequence coverage of the trout CXCR4.1a residues. (**B**): predicted local distance difference test (LDDT) score per residue for five models (model 5 = 88.9; model 3 = 88.8; model 4 = 87.2; model 1 = 85.5; model 2 = 82.6). (**C**): cartoon model of the structure of human CXCR4 and five predicted structures of trout CXCR4.1a. (**D**): prediction aligned error (PAE) score for five models. (**E**): schematic representation of interactions between human CXCR4/trout CXCR4.1a and IT1t. Red amino acids show human CXCR4 residues, and blue amino acids show trout CXCR4 residues. (**F**): sequence coverage of the trout CXCR4.1b residues. (**G**): prediction dictated local distance difference test (LDDT) score per residues for five models (model 3 = 88.8; model 4 = 88.4; model 5 = 87.2; model 1 = 84.6; model 2 = 80.1). (**H**): cartoon model of the structure of human CXCR4 and five predicted structures of trout CXCR4.1b. (**I**): prediction aligned error (PAE) score for five models. (**J**): schematic representation of interactions between human CXCR4/trout CXCR4.1b and IT1t. Red amino acids show human CXCR4 residues, and blue amino acids show trout CXCR4 residues.

Sequence coverage of trout CXCR4.1a and CXCR4.1b residues are shown in Figure 6A,F. For trout CXCR4.1a, the pLDDT score of model 5, model 3, model 4, model 1, and model 2 were 88.9, 88.8, 87.2, 85.5, and 82.6, respectively (Figure 6B, cartoon representation in Figure 6C). The pLDDT scores of model 3, model 4, model 5, model 1, and model 2 of trout CXCR4.1b were 88.8, 88.4, 87.2, 84.6, and 80.1, respectively (Figure 6G, cartoon representation in Figure 6H). Uncertainty of the predicted distance between two residues is color-coded from blue (0 Å) to red (30 Å, Figure 6D,I). Trout CXCR4.1a (CXCR4.1b) showed conserved IT1t (a small molecule antagonist) binding sites of W99 (W96), D102 (D99), W107 (W104), V117 (V114), Y121 (Y118), C193 (C188), R190 (R185), and I192 (I187) to human CXCR4 with exception of D187. Human CXCR4 D187 was replaced by Q194 in trout CXCR4.1a and Q189 in trout CXCR4.1b (Figure 6E,J).

## 4. Discussion

### 4.1. Characterization of cxcr Genes

Whole genome duplication occurred in teleost ancestors, resulting in increased paralogs of *cxcr* genes. Hence, previous studies have identified eight *cxcr* genes in a typical 3R teleost species, such as channel catfish [15]. Atlantic salmon is an important 4R salmonid species, and 19 *cxcr* genes have been identified in Atlantic salmon [61]. In the current study, a total of 17 *cxcr* genes were identified in rainbow trout based on available genomic information and our RNA-seq datasets (Figure 1 and Figure 2), being consistent with previous studies that showed an expansion of chemokine systems in teleosts [62,63]. Thus, the faster evolvement of chemokines and the fish-specific whole genome duplication have resulted in the expansion of both teleost chemokine and receptor genes [10,14,64,65,66]. In this study, we showed duplications of trout *cxcr1*, *cxcr2*, *cxcr3*, *cxcr4*, and *cxcr7* due to whole genome duplication and lineage-specific tandem gene duplications (Figure 1 and Figure 2). For example, *cxcr3* and *cxcr3.1a* were localized quite close to each other on chromosome 2, and a large cluster of *cxcr* genes were located on chromosome 3 and 22, all of this suggesting a rapid evolution through tandem duplications [67], suggesting that gene duplication of CXC chemokines and receptors might acts as a predominant evolutionary mechanism for environment adaptation in fish [68]. Compared to 3R teleosts, the 4R salmonid species exerted more *cxcr* paralogs, which is consistent with previous studies showing that genes (such as *igf* and *igfbp* genes, for example) involved in immunomodulation were further expanded in salmonid species [26,27].

The phylogenetic analysis showed explicit annotations of trout CXCR proteins, with most trout CXCR proteins clustered with their teleost counterparts (Figure 1). The seven transmembrane domains, a conserved and typical structure of GPCRs [69], were observed in all trout CXCR members, as well as a DRY motif in TM3 and an NPxxY motif in TM7, revealing sequence and structure similarities between trout CXCRs and mammalian family A GPCRs.

### 4.2. Physiological Functions of cxcr Genes

The chemokine system plays an important role in modulating the development of the immune system homeostasis during routine immune surveillance and inflammation [2,6,67]. Recent studies reported the involvement of chemokine systems in regulating immunomodulation in teleosts, including catfish, trout, croaker, and bream [15,59,70,71,72,73]. However, most of these studies focused on studying CXCR-regulated immunomodulation in peripheral immune tissues rather than in the central nervous system. Therefore, we evaluated *cxcr* transcription levels in both brain and peripheral tissues in trout in response to *V. anguillarum* or *A. salmonicida* infections.

In humans and rodents, CXCR1 has been reported to be widely expressed in the brain and to play an important role in modulating neuroinflammation [74,75]. In teleosts, CXCR1 regulates immune defense against pathogen infections [59,76,77]. For example, peripheral *cxcr1* was up-regulated by viral and bacterial infections in trout [59]. Two *cxcr1* subtypes have been identified in some teleost species as a consequence of the additional WGD [61,77], which is consistent with our results. In Asian swamp eel (*Monopterus albus*), a previous study showed different gene expressions between *cxcr1.1* and *cxcr1.2* after pathogen infection [77]. In this study, brain *cxcr1.1* and *cxcr1.2* showed different transcriptional regulation in response to bacterial infections (Figure 3). A recent study showed *cxcrs* exhibited tissue-specific and time-dependent regulation of transcription in the head kidney, liver, and gill after *A. salmonicida* infection in turbot (*Scophthalmus maximus*) and black rockfish (*Sebastes schlegelii*) [68,78]. These results suggest that *cxcr1* subtypes might be differently involved in the response to disease in both brain and peripheral tissues.

Biomedical studies indicate that CXCR3 is involved in directing lymphocytes into inflammation areas and regulating the inflammatory state of both the CNS and peripheral tissues [79,80]. A previous study in grass carp (*Ctenopharyngodon idella*) showed that *cxcr3* is widely expressed in the brain and protects the brain from pathogen infection [81]. We observed that brain *cxcr3* was significantly up-regulated by *A. salmonicida* infection (Figure 4). Consistently, transcriptional profiles of *cxcr3* subtypes were significantly altered by a bacterial and viral infection, LPS or polyI:C stimulation in teleost species, including rainbow trout, turbot, largemouth bass (*Micropterus salmoides*), and black rockfish [57,68,78,82]. In rainbow trout, *cxcr3* subtypes were differently induced by inflammatory stimulants and cytokines in head kidney cells and macrophages [59,76,83]. Our results also showed that *cxcr3* exhibited tissue-specific and subtype-dependent transcriptional regulation in peripheral tissues in response to pathogen infection. Our results further supported the involvement of CXCR3 in the neuro-immune network in fish [81]. Human studies showed that CXCR3A and CXCR3B exert opposite functions in regulating cell growth, with the “Survival” and “Death” signals derived from CXCR3A and CXCR3B, respectively [84,85]. In this study, we observed up-regulated *cxcr3* subtypes in asymptomatic trout compared to symptomatic trout (Figure 3). Our results suggested trout *cxcr3* subtypes might be functional orthologs of human CXCR3A. Further studies should investigate the regulatory mechanism(s) of the *cxcr3*-regulated “Survival” signals.

CXCR4 is one of the most well-studied chemokine receptors due to its important role in regulating the development of the immune system and also immunomodulation. In biomedical studies, CXCR4 serves as the therapeutic target of cancer metastasis and HIV-1 infection [86,87,88,89]. There is growing evidence that CXCR4 is involved in infection defense, neuron pathophysiology, and response to stress in teleosts ([90,91], Reviewed in [76]). For example, rainbow trout, grouper, and channel catfish showed up-regulated *cxcr4* after viral and bacterial infections [15,82,90]. CXCR4 was also widely expressed in the CNS, where it seems to be involved in neuron pathology [76,90]. Our results showed brain *cxcr4.1b* and *cxcr4.2b* expression were significantly altered due to *V. anguillarum* infection, in correlation with a previous study that showed that nervous necrosis virus infection led to significantly upregulated *cxcr4* expression in orange-spotted grouper [90]. Alterations of environmental nitrate also induce *cxcr4b* expression in Wuchang bream (*Megalobrama amblycephala*) [91]. Likewise, our results showed that both bacterial infection and environmental changes altered the expression of *cxcr4* subtypes (Figure 3 and Figure 5). CXCR5 serves as the homeostatic regulator for immune responses and neuron regeneration ([92,93], reviewed in [76]). In fish, *cxcr5* was shown to be highly expressed in lymphoid tissues, including the kidney and spleen in grass carp [92]. Consistent with previous studies showing *cxcr5* expression is modulated by a range of immune stimulants and pathogen infection [68,92], in this study, symptomatic trout also showed down-regulated spleen *cxcr5* in response to *V. anguillarum* infection (Figure 4).

Meanwhile, it is important to compare the extent of changes between chemokine receptors to the overall statistical pattern of changes in the RNA-Seq data and other genes that are not directly related to immunomodulation. In this study, we selected *per1b* (period circadian clock 1b). The *per1b* is widely expressed in both brain and peripheral tissues in humans (https://www.genecards.org/, accessed on 24 January 2024) and rainbow trout. The *per1b* gene regulates circadian rhythms of locomotion, metabolism, and behavior. In the brain, average fold-changes of all up-regulated genes between groups were ~1.59 (C/S) and 1.48 (C/A), and all down-regulated genes between groups were ~0.68 (C/S) and 0.75 (C/A). The fold-changes of *per1b* gene expression between groups were ~1.51 (C/S) and 0.94 (C/A), while fold-changes of *cxcr1.2* gene expression between groups were ~0.21 (C/S) and 0.57 (C/A) (Figure 3). In the kidney, average fold-changes of all up-regulated genes between groups were ~2.51 (C/S) and 1.76 (C/A), and all down-regulated genes between groups were ~0.59 (C/S) and 0.63 (C/A). The fold-changes of *per1b* gene expression between groups were ~0.84 (C/S) and 0.82 (C/A), while fold-changes of *cxcr3* gene expression between groups were ~3.97 (C/S) and 1.21 (C/A) (Figure 3). In the spleen, average fold-changes of all up-regulated genes between groups were ~4.35 (C/S) and 2.71 (C/A), and all down-regulated genes between groups were ~0.45 (C/S) and 0.60 (C/A). The fold-changes of *per1b* gene expression between groups were ~0.95 (C/S) and 1.39 (C/A), while fold-changes of *cxcr3* gene expression between groups were ~8.89 (C/S) and 0.64 (C/A) (Figure 3). These results suggested that infection or the fish response to infection are directly provoking alterations in trout *cxcr* transcription levels, especially between groups of control trout and symptomatic trout.

Finally, we showed that the amino acids of the binding pocket of IT1t (a small molecule) in trout CXCR4.1a/b were conserved to those of human CXCR4 [94] (Figure 6). Compared to endogenous ligands, these small molecule ligands (drugs) are stable and orally bioavailable [95]. Therefore, small molecular ligands of human chemokine receptors could be used in the future as immunomodulators targeting fish CXCRs in the aquaculture industry.

## 5. Conclusions

In this study, we have identified 17 *cxcr* genes in rainbow trout with duplicated copies of *cxcr1*, *cxcr2*, *cxcr3*, *cxcr4*, and *cxcr7*. Gene expression analyses showed trout *cxcr* genes exhibited conserved functions with human orthologs. Transcription levels of *cxcr* genes were altered by bacterial infection and environmental changes, suggesting a pleiotropic role in regulating homeostasis and immune response. Trout CXCR4.1a(b) showed conserved residues for the binding pocket of IT1t (a small molecule ligand) with human CXCR4. Our results contribute to a better understanding of the immune role of CXCRs and their potential ligands in an important teleost species. This information could be used in the future to modulate immune responses to infectious diseases and adaptation to environmental changes.

## Figures and Tables

**Table 1:** Summary of 17 *cxcr* genes in rainbow trout.

Gene Name	Gene ID	Chromosome	Position (bp)	Protein Length (aa)	MW (kDa)	pI	Derived from	Chemokines [10,55,56,57]	Reference
*cxcr1.1*	LOC100135914	Chr18	3,898,505–3,899,881	359	39.98	9.2	AF260964.1	CXCL8	[55,58]
*cxcr1.2*	LOC110501285	Chr22	5,736,707–5,742,123	362	40.1	8.31	Newly Identified	CXCL8	
*cxcr2.1*	LOC110520605	Chr3	78,906,276–78,908,301	362	40.3	8.94	HG794530.1	CXCL8	[55,59]
*cxcr2.2*	LOC110501383	Chr22	11,509,532–11,512,348	362	40.07	8.78	Newly Identified	CXCL8	
*cxcr3.1a*	LOC110537629	Chr2	7,072,125–7,076,959	371	41.7	6.08	Newly Identified	CXCL9, 10, 11	
*cxcr3.1b*	LOC110514317	Chr2	102–3242	373	41.94	5.88	Newly Identified	CXCL9, 10, 11	
*cxcr3b*	LOC100136126	Chr3	16,782,015–16,785,624	374	42.19	8.17	AJ888881.1	CXCL9, 10, 11	[10,55,56,59]
*cxcr3*	LOC110537622	Chr2	7,038,779–7,047,349	382	42.37	9.1	Newly Identified	CXCL9, 10, 11	
*cxcr3a*	LOC100136649	Chr3	16,764,415–16,768,463	380	42.27	9.24	AJ888878.1	CXCL9, 10, 11	[10,55,56,59]
*cxcr4.1a*	LOC110520024	Chr3	48,667,827–48,669,883	362	40.57	8.74	AJ001039.1	CXCL12	[55,60]
*cxcr4.1b*	LOC110501543	Chr22	18,785,356–18,787,371	357	39.99	8.86	Newly Identified	CXCL12	
*cxcr4.2a*	LOC110530627	Chr8	70,897,183–70,922,718	373	40.82	8.58	Newly Identified	CXCL12	
*cxcr4.2b*	LOC110516585	Chr28	1208–2756	373	41.08	8.54	Newly Identified	CXCL12	
*cxcr5*	LOC110503290	Chr24	38,512,905–38,516,651	309	33.92	5.96	Newly Identified	CXCL13	
*cxcr6*	LOC110492888	Chr16	21,961,814–21,965,417	461	50.85	8.68	Newly Identified		
*cxcr7.1a*	LOC110520437	Chr3	70,094,498–70,106,981	375	42.01	6.94	Newly Identified	CXCL11, 12	
*cxcr7.1b*	LOC110501640	Chr22	25,651,985–25,660,915	378	42.22	6.66	Newly Identified	CXCL11, 12	

**Table 2:** Comparison of *cxcr* gene copies among mammals and teleosts.

Name	Human	Mouse	Chicken	Frog	Zebrafish	Channel Catfish	Atlantic Salmon	Fugu	Northern Pike	Rainbow Trout
*cxcr1*	1 (~14%)	1 (~14%)	1 (~33%)	0	1 (10%)	1 (~11%)	2 (~11%)	2 (25%)	2 (20%)	2 (~11%)
*cxcr2*	1 (~14%)	1 (~14%)	0	0	1 (10%)	1 (~11%)	2 (~11%)	1 (12.5%)	1 (10%)	2 (~11%)
*cxcr3*	1 (~14%)	1 (~14%)	0	1 (25%)	3 (30%)	3 (~33%)	3 (~17%)	1 (12.5%)	2 (20%)	5 (~29%)
*cxcr4*	1 (~14%)	1 (~14%)	1 (~33%)	1 (25%)	2 (20%)	2 (~22%)	4 (~23%)	2 (25%)	2 (20%)	4 (~23%)
*cxcr5*	1 (~14%)	1 (~14%)	1 (~33%)	1 (25%)	1 (10%)	1 (~11%)	1 (~5%)	1 (12.5%)	1 (10%)	1 (~5%)
*cxcr6*	1 (~14%)	1 (~14%)	0	0	0	0	1 (~5%)	0	1 (10%)	1 (~5%)
*cxcr7*	1 (~14%)	1 (~14%)	0	1 (25%)	2 (20%)	1 (~11%)	4 (~23%)	1 (12.5%)	1 (10%)	2 (~11%)
Total	7	7	3	4	10	9	17	8	10	17

## Data Availability

Data are contained within the article and Supplementary Materials.

## Supplementary Materials

The following supporting information can be downloaded at https://www.mdpi.com/article/10.3390/biom14030337/s1, Text S1: Input amino acids sequences of CXCR4.1a–b, Figure S1. Prediction aligned error (PAE) score for five models with CXCR4.1a ORF sequences. Figure S2. Prediction aligned error (PAE) score for five models with CXCR4.1a amino acid sequences associated with the transmembrane domain (TM), extracellular (ECL), and intra-cellular (ICL) loops. Table S1. Count of liver *cxcr* genes in trout at HD, LD, and MD.
