# Supplementary material for: Pleiotropic Role of Rainbow Trout CXCRs in Response to Disease and Environment: Insights from Transcriptional Signatures and Structure Analysis
Source: Biomolecules. 2024 Mar 12;14(3):337. doi: 10.3390/biom14030337 (PMC10968610; doi:10.3390/biom14030337)
Supplement: Supplementary file 1 [file biomolecules-14-00337-s001.zip › biomolecules-2866510-supplementary.pdf]

Supplementary Text S1

Input amino acids sequences of CXCR4.1a
NRVGGDDFQRIFLPTVYGIIFLLGIVGNGLVVTVMGYQKKVKTMTDKYRLHL
SVADLLLVFTLPFWAVDAASSWYFGGFLCTTVHVIYTINLYSSVLILAFISVDRY
LAVVHATNSQTTRKRKLLAERWIYVAVWLPAAVLTVPDIVFATALDGGSRTICQ
RIYPQKTSFYWMAGFRFQHILVGFVLPGLVILTCYCIIIAKLSQGAKGQVLKRK
ALKTTVILILCFFSCWLPYCVGIFLDTLMLLNVISHSCALEQSLQTWLLITEAL
AYFHCCLNPILYAFL

Input amino acids sequences of CXCR4.1b
NRVSGDDFQRIFLPTVYGIIFLLGIVGNGLVLIVMGYQKKVQTKTDKYRLHLS
VADLLFVLTLPFWAVDAASSWYFGGILCTAVHVIYTINLYSSVLILAFISMDRYL
AVVHATKSQSTRTFLADRVIYVAVWLPAVILTVPDMVFATAQNRVSRTICQRIYP
QETSFYWMAGFRFQHILVGFVLPGLVILTCYCIIIAKLSQGSKGQVLKRKALKT
TVILVLCFFSCWLPYCVGIFVDTLMLLNVISHSCALEQSLQTWISITEALAYFHC
CLNPILYAFL

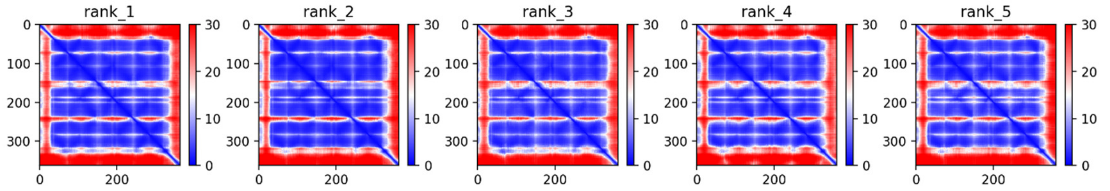

Figure S1. Prediction aligned error (PAE) score for five models with CXCR4.1a ORF sequences

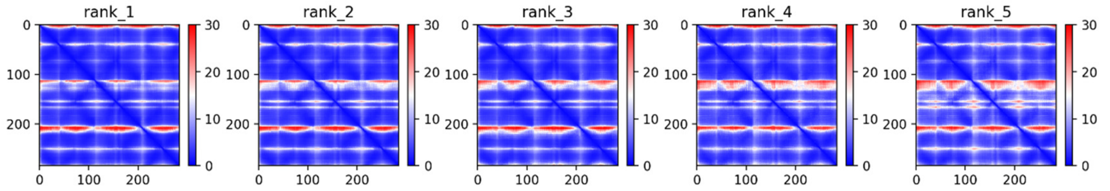

Figure S2. Prediction aligned error (PAE) score for five models with CXCR4.1a amino acid sequences associated with transmembrane domain (TM), extracellular (ECL) and intra-cellular (ICL) loops.

Table S1. Count of liver *cxcr* genes in trout at HD, LD and MD.

gene	*HD_1*	*HD_2*	*HD_3*	*LD_1*	*LD_2*	*LD_3*	*MD_1*	*MD_2*	*MD_3*
cxcr1.1	4	16	11	2	8	5	14	9	4
cxcr1.2	4	7	1	0	0	4	1	0	1
cxcr2.1	4	1	2	0	0	0	0	1	0
cxcr2.2	0	1	0	0	0	0	0	1	0
cxcr3	10	6	24	11	14	10	8	18	19
cxcr3.1a	0	3	3	1	3	0	4	3	7
cxcr3.1b	5	5	16	5	6	6	2	9	5
cxcr3a	32	24	64	97	52	64	59	56	48
cxcr3b	0	2	1	0	2	1	0	0	2
cxcr4.1a	2	7	1	5	5	4	7	3	3
cxcr4.1b	16	11	15	14	15	7	11	10	8
cxcr4.2a	4	3	5	5	2	2	7	1	10
cxcr4.2b	2	0	3	6	2	3	3	10	2
cxcr5	1	6	3	2	4	2	5	2	4
cxcr6	0	0	0	0	1	0	0	0	0
cxcr7.1a	0	0	0	0	0	0	0	0	0
cxcr7.1b	153	111	181	177	560	55	208	107	489
